# Influence of Deposition Method on the Structural and Optical Properties of Ge_2_Sb_2_Te_5_

**DOI:** 10.3390/ma14133663

**Published:** 2021-06-30

**Authors:** Iosif-Daniel Simandan, Florinel Sava, Angel-Theodor Buruiana, Aurelian-Catalin Galca, Nicu Becherescu, Ion Burducea, Claudia Mihai, Alin Velea

**Affiliations:** 1 National Institute of Materials Physics, Atomistilor 405A, 077125 Magurele, Romania; simandan@infim.ro (I.-D.S.); fsava@infim.ro (F.S.); angel.buruiana@infim.ro (A.-T.B.); ac_galca@infim.ro (A.-C.G.); claudia.mihai@infim.ro (C.M.); 2 Apel Laser Ltd., Vanatorilor 25, 077135 Mogosoaia, Romania; becherescu@gmail.com; 3 Horia Hulubei National Institute of Physics & Nuclear Engineering, 077125 Magurele, Romania; bion@nipne.ro

**Keywords:** magnetron sputtering, pulsed laser deposition, Ge2Sb2Te5, Raman spectroscopy, spectroscopic ellipsometry, band gap

## Abstract

Ge2Sb2Te5 (GST-225) is a chalcogenide material with applications in nonvolatile memories. However, chalcogenide material properties are dependent on the deposition technique. GST-225 thin films were prepared using three deposition methods: magnetron sputtering (MS), pulsed laser deposition (PLD) and a deposition technique that combines MS and PLD, namely MSPLD. In the MSPLD technique, the same bulk target is used for sputtering but also for PLD at the same time. The structural and optical properties of the as-deposited and annealed thin films were characterized by Rutherford backscattering spectrometry, X-ray reflectometry, X-ray diffraction, Raman spectroscopy and spectroscopic ellipsometry. MS has the advantage of easily leading to fully amorphous films and to a single crystalline phase after annealing. MS also produces the highest optical contrast between the as-deposited and annealed films. PLD leads to the best stoichiometric transfer, whereas the annealed MSPLD films have the highest mass density. All the as-deposited films obtained with the three methods have a similar optical bandgap of approximately 0.7 eV, which decreases after annealing, mostly in the case of the MS sample. This study reveals that the properties of GST-225 are significantly influenced by the deposition technique, and the proper method should be selected when targeting a specific application. In particular, for electrical and optical phase change memories, MS is the best suited deposition method.

## 1. Introduction

Ge–Sb–Te alloys are intensely studied and used for optical and electrical memory applications due to their fast and reversible crystalline to amorphous phase transition that leads to a remarkable change in reflectivity and resistivity [1,2,3]. GST-225 has enabled the development of phase-change random access memories and can be integrated in nonvolatile memory structures, which have better scaling capabilities when compared to flash memory [4]. This has led to a large number of studies on their structural and optical properties [5,6,7].

Issues with the crystallization speed, resistance drift and optical contrast of GST-225 are hindering its use in next generation memory devices. Thus, the improvement of its structural and optical properties is crucial for the development of faster, denser, low-power and enduring nonvolatile memories.

GST-225 can be transformed between amorphous, metastable face-centered-cubic (fcc) and stable hexagonal close-packed (hcp) phases. The first transition, near 150 °C, is from the amorphous to fcc phase, while the second transition, above 230 °C, is from the fcc to hcp phase [3]. The crystalline phase of GST-225 is more optically reflective and more electrically conductive than the amorphous phase. 

The fcc metastable GST-225 (or (Ge_0.4_Sb_0.4_)Te) phase is a NaCl structure, with the space group Fm-3m, where Te occupies the anion positions, while Ge, Sb and vacancies randomly occupy the cation sites. The concentration of vacancies is approximately 20 at. % for the stoichiometric compound and varies with composition [8]. Even if this phase is metastable, it can be robust at room temperature for decades [8]. On the other hand, the stable hcp crystalline phase of GST-225 is a hexagonal structure with atomic alternating blocks of Ge/Sb and Te layers held together by van der Waals forces [9].

Phase change materials are known to exhibit deposition-dependent optical and structural properties, such as mass density, optical bandgap and refractive index [10]. GST-225 films have been successfully obtained through a wide variety of deposition techniques such as magnetron sputtering [11], pulsed laser deposition [12], atomic layer deposition [13], chemical vapor deposition [14], etc.

Magnetron sputtering (MS) is a thin film deposition technique that is widely used to obtain uniform and smooth films [15]. Energetic ions of argon, from the plasma of a gaseous discharge inside a vacuum chamber, are accelerated by an electric field to come into collision with a target made of the material to be sputtered. With the help of a magnetic field, the electrons in the plasma are confined near the surface of the target. By confining the electrons, a higher density plasma is obtained and therefore an increased deposition rate of the sputtered material [16]. MS has the advantage that it can be used to obtain very large area film coatings. It also offers other advantages compared to thermal or e-beam deposition techniques, since the more energetic atoms obtained by magnetron sputtering can improve the adhesion of the deposited film [15].

Pulsed laser deposition (PLD) is a thin film deposition technique in which an intense laser pulse enters through an optical window of a vacuum chamber and is focused onto a target. Depending on the target material, above a certain power density, the ablation process occurs in the form of a plume, where the ejected material from the target is directed by the plume to a desired substrate [17]. PLD, compared to other thin film deposition methods, has the main advantage that under optimal conditions, the ratios of the elemental components of the target and those of the deposited thin film are almost identical, even for ternary (or higher order) targets [18,19]. Without a doubt, the most important drawback of PLD is the droplet production on the substrate that occurs during deposition when the mechanical forces created by the laser power acting on the target lead to the ejection of microscopic particulates [20,21].

There have been several reports combining MS and PLD (MSPLD) for the deposition of thin films for materials such as carbides and diamond-like carbon films [22], ternary wolfram borides [23] or tin oxide films [24]. The advantages of the combined MS and PLD deposition is a smoother surface [24] and a denser and higher hardness of the obtained films [23].

The objective of this work is to compare the structural and optical properties of GST-225 films obtained by magnetron sputtering, pulsed laser deposition and a deposition technique combining both methods. In the combined deposition, the formation of the film takes place simultaneously from the same target with the help of two plasma fluxes that are different in energy content and particle density. One might expect to obtain thin films which have the advantages of both MS and PLD, namely improved structural and optical properties. To our knowledge, this is the first time that GST-225 thin films have been obtained using combined MS and PLD.

## 2. Materials and Methods

GST-225 thin films were prepared at room temperature on glass and silicon substrates (two substrates with a size of 12 × 12 mm^2^ were used for each deposition).

The first sample set was deposited by MS at an argon pressure of 5 × 10^−5^ Torr (Linde, Bucharest, Romania). To sputter the GST-225 target (Mateck GmbH, Jülich, Germany), a Model AG 0313 source (RF T&C Power Conversion Inc., New York, NY, USA) and a 3G Circular Magnetron (Gencoa, Liverpool, UK) were used. Prior to deposition, by using the Q-pod calibration software (Inficon, Bad Ragaz, Switzerland) connected to a quartz crystal, a deposition rate of 1 Å/s for a 12 W power was obtained. The substrates were placed at a distance of 8 cm from the 2 in targets and the substrate holder was continuously rotated in order to obtain a uniform deposition of ~100 nm during 1000 s deposition time.

Another set of GST-225 films were prepared by PLD. A COMPexPro KrF laser source (Coherent, Santa Clara, CA, USA) with a 248 nm wavelength and 1.5 J/cm^2^ fluence at a 45° incidence angle on the target was used. During calibration, in order to obtain a 1 Å/s deposition rate, a 5 Hz repetition rate and a laser power of 110 mJ were utilized.

A final set of GST-225 films were deposited by MSPLD (a picture of the custom-built equipment is shown in Supplementary Figure S1) using the above-described parameters for deposition. The obtained deposition rate for MSPLD was 2 Å/s, and the deposition time was adjusted to 500 s in order to obtain film thicknesses of ~100 nm.

The three types of GST-225 thin films were annealed in vacuum (7 × 10^−2^ Torr) at 200 °C for 1 h using a GSL 1600X furnace (MTI, Richmond, CA, USA) with an increment of 1.5 °C per min for heating and cooling.

For sample composition, Rutherford backscattering spectrometry (RBS) experiments, using alpha particles delivered by a 3 MV Tandetron (High Voltage Engineering Europa B.V., Amersfoort, The Netherlands) [25], were performed. The RBS spectra were simulated using the SIMNRA^®^ software package version 7.0 (developed by Matej Mayer at the Max-Planck-Institut für Plasmaphysik, Garching, Germany) [26].

The thin film structure was investigated by grazing incidence X-ray diffraction (GIXRD) at an incidence angle of 0.4° using a Rigaku SmartLab diffractometer (Rigaku, Tokyo, Japan) equipped with HyPix-3000 2D Hybrid Pixel Array Detector (Rigaku, Tokyo, Japan) in “0D” mode. The identification of the crystalline phases was performed with the help of DIFFRAC.SUITE Software package version 4.2 (Bruker AXS GmbH, Karlsruhe, Germany). With the same equipment, the X-ray reflectometry (XRR) data were registered in the range 0–3° (2θ), with the step of 0.004° (2θ). In order to obtain the mean thickness and the mean mass density of each thin film, LEPTOS software version 3.04 (Bruker AXS GmbH, Karlsruhe, Germany) was used to fit a simulated XRR curve to the measured XRR data. 

The Raman spectra were acquired at room temperature, in the 60–200 cm^−1^ range in backscattering configuration, with a LabRAM HR Evolution spectrometer (Horiba Jobin-Yvon, Palaiseau, France) provided with a confocal microscope. A He–Ne laser (Horiba Jobin-Yvon, Palaiseau, France) operating at 633 nm was focused using an Olympus 100x objective (Olympus, Tokyo, Japan) on the surface of thin films.

The spectroscopic ellipsometry measurements were made using a Woollam Vertical-Variable Angle Spectroscopic Ellipsometer (J.A. Woollam Co., Lincoln, NE, USA) at angles of incidence (AOI) of 55 and 65°. The range of the used wavelength was between 0.7 and 3 eV. The WVASE32 software was used to evaluate the optical parameters of the films.

## 3. Results and Discussion

### 3.1. Rutherford Backscattering

The composition of the GST-225 films has been measured by RBS. The determined parameters, stoichiometry and thickness are presented in Table 1. As expected, the stoichiometry of the PLD film is the closest to the ideal GST-225 (i.e., 22.2 at. % of Ge, 22.2 at. % of Sb and 55.6 at. % of Te), while for the other two methods, a small amount of Te is lost during deposition. The results can be affected by errors for Sb and Te, with errors up to 10–15%. This is because Sb and Te cannot be energetically separated for the maximum energy used of 4.28 MeV, while for Ge, the error is smaller than 5%. For the films deposited by PLD and MSPLD, a pronounced diffusion effect of the Sb and Te atoms towards the substrate is evidenced. For the film deposited by MS, the diffusion effect is much smaller. This is due to the fact that in the case of PLD, the energy of the particles in the plasma is higher, whereas the Sb and Te atoms are much heavier than the Ge atoms. Thickness measurements from RBS are expressed in thin film units (TFU), where 1 TFU = 10^15^ atoms/cm.

### 3.2. X-ray Reflectivity

**Figure 1:**
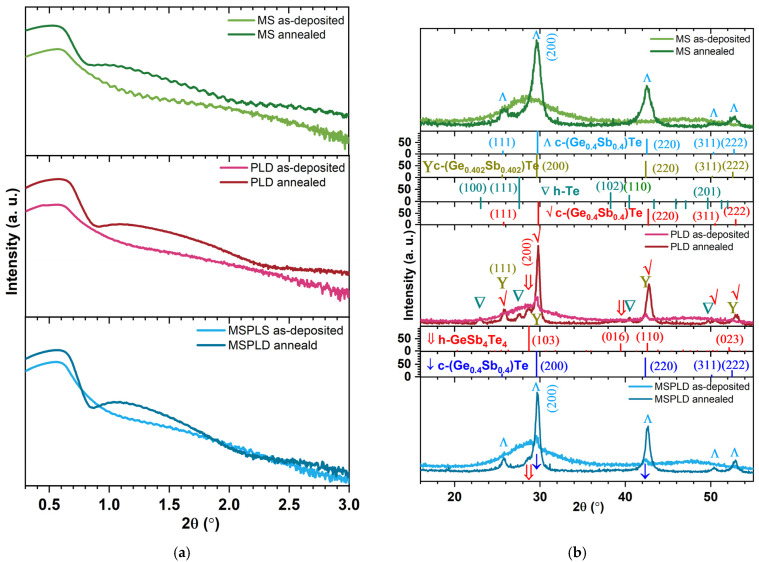
X-ray reflectometry (XRR) diagrams (**a**) and Grazing incident X-ray diffraction (GIXRD) patterns (**b**) for thin films of GST-225 deposited by: magnetron sputtering, pulsed laser deposition and simultaneously using the two methods in as-deposited states and after annealing at 200 °C.

Fitting a simulated XRR curve (not shown) to the measured XRR data (Figure 1a) of the three types of GST-225 thin films (deposited by magnetron sputtering, pulsed laser deposition and simultaneously using the two methods) in the as-deposited state and after annealing at 200 °C, the mean mass density (*ρ*_m_) and the average thickness (h_m_) of the thin films (see Table 2) are obtained.

The dynamical Parratt formalism is integrated in LEPTOS, which allows for the calculation of the X-ray reflectivity from a multilayer sample model created by the user on a trial-and-error approach. Among the sample model’s parameters in LEPTOS are the thickness (h_i_) and the mass density (*ρ*_i_) of each layer. The calculated and measured reflectivities are compared, and automated optimization procedures (fitting) are used to minimize their difference by exploring the space of the sample parameters. A measure for the agreement between simulation and experimental data is the cost function (χ^2^). If χ^2^ is big, another multilayer sample model is created (even though the sample is a single thin film, the final sample model consists of five layers), and fitting is resumed. For the final sample models, χ^2^ ranges between 3 × 10^−4^ and 2 × 10^−3^, which are very good values. The total thickness determination accuracy is ±5% (the error is ±5 nm), while that of the total mass density is ±2% (the error is ±0.12 g/cm^3^).

In the as-deposited state, the highest value of the *ρ*_m_ (6.13 ± 0.12 g/cm^3^) is obtained for the MS deposited GST-225 thin film, while in the annealed state, it is for the MSPLD thin film (6.42 ± 0.12 g/cm^3^). According to Njoroge et al. [27], this high value corresponds to the formation of nearly void-free films. The highest change in mass density is observed in PLD films, an increase of 9.5%, which is higher than the usual change in density that accompanies the transition from a-GST-225 to c-GST-225 of 6.5% [28]. For MS films, however, the change in density is the smallest and is ideal for electronic memory devices. Usually, a high percentage of vacancies contributes to the suppression of volume change during the transition from the amorphous to the crystalline phase [28].

### 3.3. X-ray Diffraction

The grazing incident X-ray diffraction (GIXRD) patterns of the three types of GST-225 thin films in the as-deposited state and after their annealing at 200 °C are shown in Figure 1b. Their atomic structure is summarized in Table 3.

It can be observed that among the samples in the as-deposited state, the only fully amorphous is the MS film. The other two samples (PLD and MSPLD) contain a small polycrystalline phase which is probably due to the PLD particulates, as was also observed earlier in chalcogenide materials obtained by PLD [29]. After annealing, fcc-GST-225 is obtained as a major phase in all the samples, the differences (as shown in the estimation of mass densities from XRR) being given by the value of the lattice constant (*a*, if *a* is smaller, density is bigger) and by the voids’ volume between crystallites. The MS sample is not completely crystalline: it has 18% amorphous phase left. It was shown that the films obtained by magnetron sputtering crystallize at a higher temperature than in the case of PLD [10,30]. On the other hand, in the other two samples (PLD and MSPLD), a minor hexagonal GeSb_4_Te_4_ is obtained, which can be regarded as a seed for the hcp-GST-225 formation at a higher annealing temperature [3]. Moreover, the segregation of hexagonal Te is obtained, which was also identified in other chalcogenide thin films when using PLD [10].

Comparing the crystallites’ average size of the main c-GST-225 phase in each annealed sample (see Table 3), the results show that MS produces a finer sputtering (and as a consequence, a larger compositional disorder) than the PLD method. Another reason could be the fact that the excess Sb from the MS sample, observed by RBS, does not enter the Ge_2_Sb_2_Te_5_ lattice to fill up the empty sites but remains at the grain boundary producing the decrease of the average grain size [28].

### 3.4. Raman Spectroscopy

**Figure 2:**
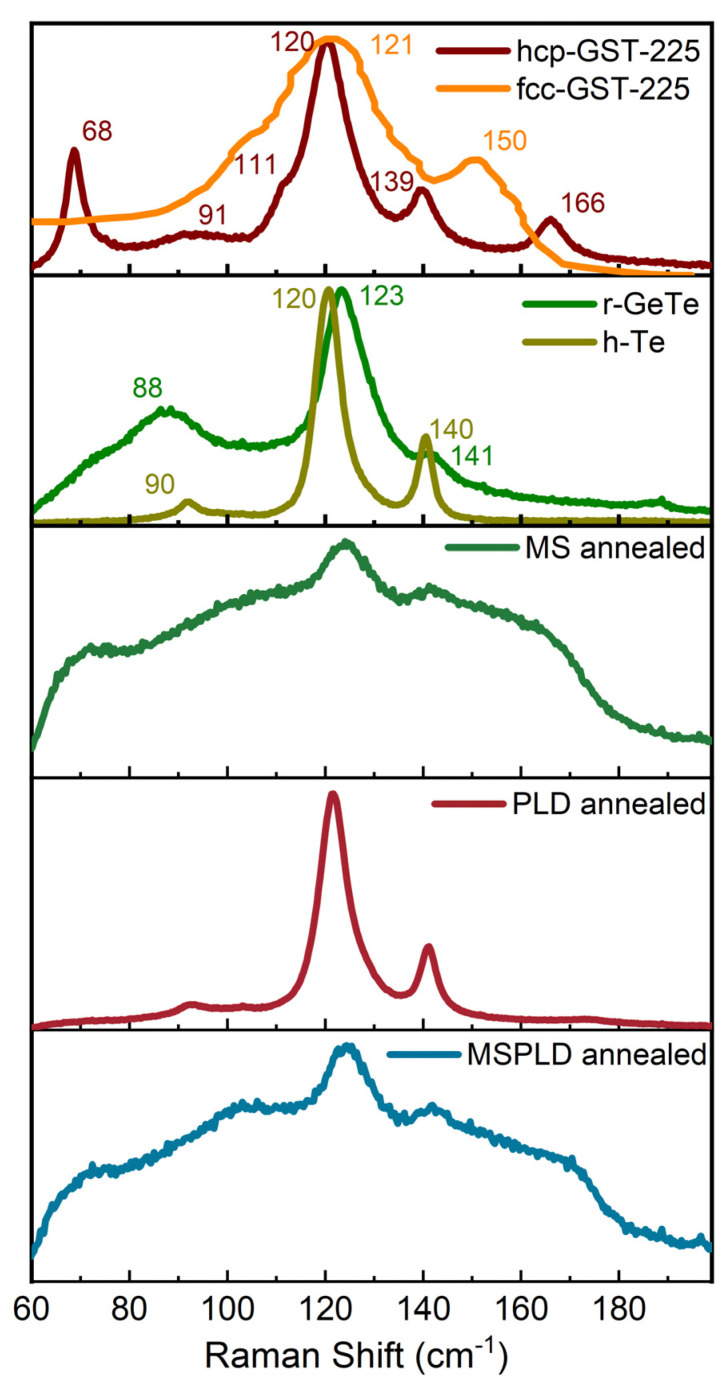
Raman spectra obtained on MS, PLD and combined MSPLD samples. fcc-GST-225 [34], hcp-GST-225, r-GeTe and h-Te were also added for comparison.

In the last decade, there has been significant progress made in the structural characterization of GST-225 by employing Raman spectroscopy [31,32,33]. The Raman spectra measured on the annealed films are presented in Figure 2. For peak identification, the spectra of hcp-Ge_2_Sb_2_Te_5_, h-Te and r-GeTe measured on bulk samples and fcc-Ge_2_Sb_2_Te_5_ [34] were also added to the figure. The polycrystalline hexagonal bulk GST-225 spectrum presents the bands at 68, 91, 120, 139 and 166 cm^−1^ and a peak shoulder at 111 cm^−1^, while that of the rhombohedral bulk GeTe shows only two peaks at 123 and 141 cm^−1^ and a broad band at 88 cm^−1^. The two prominent GeTe peaks have been assigned to the E^2^_g_ and A_1g_, respectively. The E^2^_g_ mode corresponds to the atoms vibrating in the basal plane, while the A_1g_ mode corresponds to the atoms oscillating along the c-axis. These modes are affected by the forces between Ge and Te atoms [35]. The fcc-Ge_2_Sb_2_Te_5_ shows a peak at 120 cm^−1^ and another at ~150 cm^−1^ that could be related to the peak at 168 cm^−1^ [34]. The first Raman peak of h-Te located at 90 cm^−1^ has been assigned to the E^1^ mode, the second one located at 120 cm^−1^ has been assigned to the A^1^ mode and the last one at 140 cm^−1^ has been assigned to the E^2^ mode [36].

Since GST-225 can be obtained by combining the Ge-Te and Sb-Te alloys, the spectrum can be explained by comparing the GST-225 phonon modes to those of binary alloys. Therefore, the peak at 68 cm^−1^ is linked with the bending mode of GeTe_4_ [37]. The two modes that are also present in the GeTe spectra, E^2^_g_ and A_1g_, which in this case are caused by the vibrations of Ge–Te and Sb–Te atoms in opposite directions, are shifted due to the interatomic forces [35]. The peak-shoulder at 111 cm^−1^ can be assigned to the vibration of the hetero-polar bond in tetrahedral GeTe_4_ and/or pyramidal SbTe_3_ [38]. The peaks at ~90 cm^−1^ have been assigned to the Γ_3_(E) mode and have been experimentally evidenced in single-crystalline GeTe [39].

Nemec et al. [33] have done Raman spectroscopy on hcp-GST-225 and r-GeTe bulk materials, except for the 50–100 cm^−1^ region, the peak at 166 cm^−1^ being absent in their work. However, this was observed by others [32,37,40], although the origin is still controversial. From the present measurements, it can be stated that this peak involves only Sb-related bonds, the assignment of Vinod et al. [38] thus being the correct one. One should note the most intense peak has different Raman shifts for the two compounds. 

The film obtained by PLD shows a maximum in between the two mentioned absolute maxima of hcp-GST-225 and r-GeTe, but it is very similar to h-Te. Note that the same values and shifts were reported on GeTe and GST-225 nanowires [35]. On the other hand, the fcc-GST-225 phase presents only two Raman modes [41], as in the present case, suggesting that the main phase obtained is the fcc one. Since the XRD shows the presence of a single element phase (hexagonal Tellurium), measurements on h-Te have also been added to the plot (Figure 2). As can be seen, the Raman shifts (also reported previously by [36]) of the peaks are comparable to those of r-GeTe and cubic GST-225. Thus, it is difficult to identify the aforementioned phases by Raman spectroscopy.

The annealed samples deposited by MS and MSPLD show large Raman peaks due to the fact that they have not been fully crystallized, or the crystallites are very small, as was also observed in XRD. The main peaks at 123 and 141 cm^−1^ show that fcc-GST-225 was formed in these samples.

### 3.5. Spectroscopic Ellipsometry

**Figure 3:**
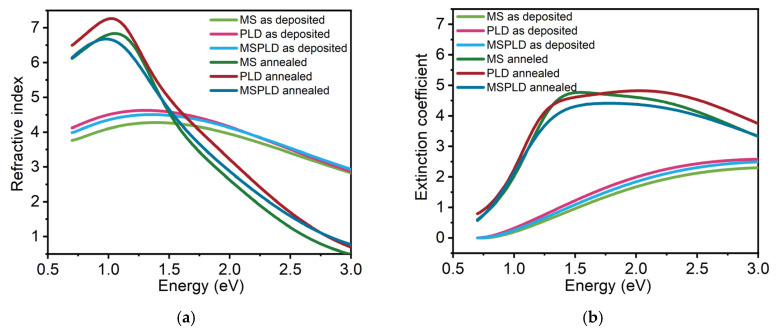
Comparison of optical functions of GST-225 films deposited by three deposition techniques. (**a**) Refractive index. (**b**) Extinction coefficient.

Optical spectroscopy has proven to be a good investigation technique for studying the structure of amorphous and crystalline materials. The refractive index and extinction coefficient as a function of wavelength, in both amorphous and crystalline phases, are important parameters in phase change materials. The results of the ellipsometry measurements are presented in Figure 3 and summarized. The fitting of the as-deposited GST-225 films was performed using a three-layer model of optical functions composed from the glass substrate, the chalcogenide thin film and the surface layer. For the as-deposited chalcogenide films, the Tauc-Lorentz (TL) dispersion formula was employed. The TL expression was developed by Jellison and Modine [42] and serves as the most widely used characterization for the optical functions of amorphous semiconductors. For the annealed GST-225 films, TL was improved by implementing an additional Gaussian oscillator to compute the optical function spectra of the crystalline phase [43].

The plotted curves obtained for the film deposited by MSPLD have a similar shape to those obtained for MS and PLD of the as-deposited GST-225. The refractive indices at 405 nm (the wavelength used for BD), at 587.6 nm (visible region), at 650 nm (the wavelength used for DVD-RAM), at 780 (the wavelength used for CD-RW) and at 1550 nm (near-IR region) [44] are shown in Table 4. A slightly smaller refractive index (3.84) for the MS compared to PLD and MSPLD films near the center of the visible domain (587.6 nm) can be observed. For PLD, the values of the refractive index and extinction coefficient are comparable to those presented by Nemec et al. [33].

In the case of the GST-225 annealed films, which present a polycrystalline structure, the refraction index of all three different types of films had an increase in values between 0.7 and 1.5 eV compared to the as-deposited films and a decrease after 1.5 eV. The refractive index increases by 65% in MS samples, at 1550 nm, and has a maximum of 6.78 at 1 eV in the PLD film at this wavelength.

The MSE values show the goodness of fit. These values are low except for the annealed MS sample, which is not entirely crystalline, and the optical model fails to fully describe both phases. The thicknesses of all the GST-225 films were in the range of 100–140 nm. The determined thickness for the MS is 103 nm and for PLD is 118 nm, whereas the thickness for the MSPLD film rises up to 141 nm. These values are in agreement with the thicknesses obtained from XRR measurements, suggesting that the model correctly describes the films in the as-deposited state. After crystallization, the thickness of all the films decreases, correlated with the increase of mass density observed in X-ray reflectivity measurements.

Inspecting the results presented in Table 5, one can observe that the Lorentz oscillator amplitude A, the oscillator resonance energy *E*_0_, the oscillator width *Γ* and the optical band gap energy *E_g_^opt^* decrease due to the phase change in the GST-225 layers. An *E_g_^opt^* of 0.75 eV for the MS films, 0.69 eV for PLD and 0.72 eV for MSPLD have been obtained. After annealing, the band gaps decrease to 0.39, 0.63 and 0.54 eV, respectively. Except for the MS film, the other values are in agreement with the values obtained by Němec et al. [33]. Bong-Sub Lee and John R. Abelson [45] have determined for the amorphous GST-225 films an *E_g_* of 0.7 eV, which is very similar to the ones determined in this study. However, in the case of the MS film, the bandgap is in accordance with the value found by Feng Rao et al. [46] who have also annealed the GST-225 films at ~200 °C and found a value of 0.36 eV for the optical band gap.

The decrease in the optical band gap after annealing is correlated with the decrease in resonance energy, which is the transition energy from the valence band of the lone pair state to the conduction band in chalcogenide materials [47]. On the other hand, the decrease in the broadening parameter Γ which shows the variations in bond lengths, bond angles and chemical disorder is consistent with the transition from the disordered amorphous state to the ordered crystalline state [33].

**Figure 4:**
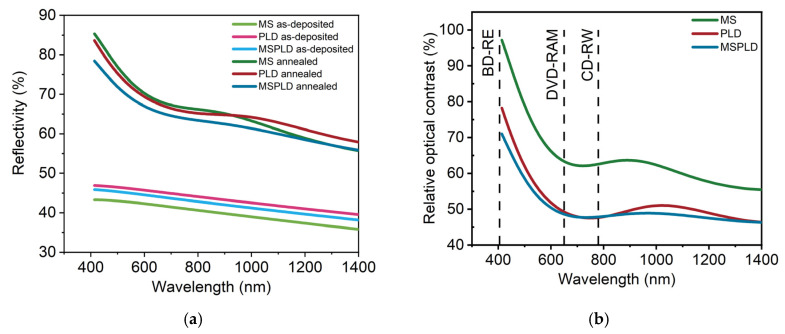
Optical reflectivity (**a**) and Relative optical contrast (**b**) for MS, PLD and MSPLD GST-225 films. The wavelengths of interest for CD-RW, DVD-RAM and BD-RE, namely 405, 650 and 780 nm, are evidenced by dashed lines.

Important parameters for optical memories are the optical reflectivity and the relative optical contrast, computed as in [48], which is needed to obtain a high signal-to-noise ratio. The reflectivity of the films in the as-deposited state is between 36 and 47%, whereas after annealing, the reflectivity is between 55 and 85% in the measured wavelength range (Figure 4a). The highest relative optical contrast is observed in Figure 4b for magnetron sputtering. The main reason for this superior contrast is the single-phase crystallization of MS films as compared with PLD and MSPLD films. As previously observed [49], disorder vacancies in the crystalline phase and mixed crystalline phases can influence the reflectivity.

## 4. Conclusions

In this paper an investigation of the influence of the deposition method on the structural and optical properties of GST-225 was reported.

In terms of film composition, PLD leads to the best stoichiometric transfer. Regarding the structure of the as-deposited films, MS produced a fully amorphous film while PLD and MSPLD present a small polycrystalline phase. On the other hand, the structure of the annealed films is of a single crystalline phase in MS films, whereas for PLD and MSPLD, there are multiple phases present. MS films have the highest optical contrast between the as-deposited and annealed state. The annealed MSPLD films have the highest mass density, while the MS films have the smallest density variation between the as-deposited and annealed states. All three methods have produced films with a similar optical bandgap of approximately 0.7 eV. Two Raman bands at 123 and 141 cm^−1^ are evidenced for the MS and MSPLD films corresponding to the fcc-GST-225. 

In conclusion, the properties of GST-225 are significantly influenced by the selected deposition technique. The appropriate method should be selected when targeting a specific application. In nonvolatile memories, for the best performance, one needs to have a material with a stable amorphous phase which switches into a single stable crystalline phase and a high resistivity and optical contrast between the two states. Additionally, a small density variation is needed when integrating the material into memory devices. The study shows that for electrical and optical phase change memories, MS is the best suited deposition method.

## Figures and Tables

**Table 1:** Composition of the thin films obtained by RBS.

Deposition Method	Thickness (TFU)	Stoichiometry (%)		
		Ge	Sb	Te
MS	333	22.6	30.5	46.9
PLD	410	20.6	23.7	55.7
MSPLD	422	21.1	27.9	51.0

**Table 2:** The mean mass density (*ρ*_m_ ± 0.12 g/cm^3^) and the average thickness (h_m_ ± 5 nm) of the GST-225 thin films.

Deposition Method	*ρ*_m_ (g/cm^3^)		h_m_ (nm)	
	As-Deposited	Annealed at 200 °C	As-Deposited	Annealed at 200 °C
MS	6.13	5.96	103.0	102.4
PLD	5.48	6.00	133.1	128.8
MSPLD	6.00	6.42	143.7	136.7

**Table 3:** Crystalline phases in GST-225 thin films. The average size of the crystallites was approximated using Scherrer equation for (200) c-GST-225 peak.

	As-Deposited	Annealed at 200 °C
MS	100% amorphous phase	- 82% polycrystalline cubic (Ge_0.4_Sb_0.4_)Te, space group Fm-3m (225), ICDD file 01-076-7107. The average size of the crystallites: 8 nm. - 18% amorphous phase
PLD	- 93% amorphous phase - 7% polycrystalline cubic (Ge_0.402_Sb_0.402_)Te, space group Fm-3m (225), ICDD file 01-078-3710.	- (85%) polycrystalline cubic (Ge_0.4_Sb_0.4_)Te, space group Fm-3m (225), ICDD file 04-011-9024. The average size of the crystallites: 63 nm. - (5%) polycrystalline hexagonal GeSb_4_Te_4_, space group P-3m1 (164), ICDD file 04-018-6310. - (10%) polycrystalline hexagonal Te, space group P3121 (152), ICDD file 00-036-1452.
MSPLD	- 94% amorphous phase - 6% polycrystalline cubic (Ge_0.4_Sb_0.4_)Te, space group Fm-3m (225), ICDD file 00-054-0484.	- 90% polycrystalline cubic (Ge_0.4_Sb_0.4_)Te, space group Fm-3m (225), ICDD file 01-076-7107. The average size of the crystallites: 36 nm. - 5% polycrystalline hexagonal GeSb_4_Te_4_, space group P-3m1 (164), ICDD file 04-018-6310. - 5% amorphous phase.

**Table 4:** Refractive indices (±0.03) of the as-deposited and annealed Ge_2_Sb_2_Te_5_ thin films.

Wavelength (nm)	Refractive Index					
	MS As-Deposited	MS Annealed	PLD As-Deposited	PLD Annealed	MSPLD As-Deposited	MSPLD Annealed
405	2.82	0.48	2.87	0.69	2.93	0.77
587.6	3.84	2.29	4.02	2.86	4.01	2.56
650	4.03	2.90	4.25	3.51	4.22	3.14
780	4.23	4.08	4.53	4.61	4.45	4.26
1550	3.87	6.38	4.27	6.78	4.12	6.42

**Table 5:** Best fit model parameters with errors of the studied Ge_2_Sb_2_Te_5_ thin films.

	MS As-Deposited	MS Annealed	PLD As-Deposited	PLD Annealed	MSPLD As-Deposited	MSPLD Annealed
MSE	5.98	11.99	6.72	7.86	6.55	6.33
A	109.3 ± 0.89	54.83 ± 4.27	123.97 ± 1.17	82.79 ± 6.25	121.56 ± 0.86	89.89 ± 4.6
*E*_0_ (eV)	2.49 ± 0.01	1.28 ± 0.01	2.39 ± 0.02	1.17 ± 0.1	2.49 ± 0.01	1.22 ± 0.01
Γ (eV)	3.72 ± 0.02	0.69 ± 0.04	3.63 ± 0.02	0.60 ± 0.01	3.78 ± 0.02	0.89 ± 0.01
*E_g_^opt^* (eV)	0.75 ± 0.01	0.39 ± 0.06	0.69 ± 0.005	0.63 ± 0.02	0.72 ± 0.01	0.54 ± 0.02
E_G_ (eV)		1.73 ± 0.05		1.65 ± 0.01		1.71 ± 0.01
A_G_ (eV)		17.71 ± 1.72		28.31 ± 0.66		15.71 ± 0.44
Γ_G_ (eV)		1.31 ± 0.06		1.55 ± 0.01		1.49 ± 0.015
Thickness (nm)	102.83 ± 0.11	69.07 ± 0.88	117.91 ± 0.14	98.29 ± 0.89	141.134± 0.12	118.76 ± 0.79

MSE—mean square error. A—Lorentz oscillator amplitude. *E*_0_—resonance energy. Γ—oscillator width. *E_g_^opt^*—optical band gap energy. E_G_ (eV)—resonance energy (Tauc–Lorentz and Gaussian oscillator (TL + G_osc_) model). A_G_ (eV)—oscillator amplitude (TL + G_osc_). Γ_G_—oscillator width (TL + G_osc_).

## Data Availability

The data presented in this study are available on a reasonable request from the corresponding author.

## Supplementary Materials

The following are available online at https://www.mdpi.com/article/10.3390/ma14133663/s1, Figure S1: The custom built MSPLD deposition system.
