# Supplementary material for: Influence of Deposition Method on the Structural and Optical Properties of Ge2Sb2Te5
Source: Materials (Basel). 2021 Jun 30;14(13):3663. doi: 10.3390/ma14133663 (PMC8269865; doi:10.3390/ma14133663)
Supplement: Supplementary file 1 [file materials-14-03663-s001.zip › materials-1277367-supplementary.pdf]

materials

Supplementary Material

Influence of Deposition Method on the Structural and Optical Properties of $Ge_2Sb_2Te_5$

Iosif-Daniel Simandan [1], Florinel Sava [1], Angel-Theodor Buruiana [1], Aurelian-Catalin Galca [1], Nicu Becherescu [2], Ion Burducea [3], Claudia Mihai [1] and Alin Velea [1,*]

[1] National Institute of Materials Physics, Atomistilor 405A, 077125 Magurele, Romania; simandan@infim.ro (I.-D.S.); fsava@infim.ro (F.S.); angel.buruiana@infim.ro (A.-T.B.); ac_galca@infim.ro (A.-C.G.); claudia.mihai@infim.ro (C.M.)
[2] Apel Laser Ltd., Vanatorilor 25, 077135 Mogosoaia, Romania; becherescu@gmail.com
[3] Horia Hulubei National Institute of Physics & Nuclear Engineering, 077125 Magurele, Romania; bion@nipne.ro
* Correspondence: alin.velea@infim.ro

Citation: Simandan, I.-D.; Sava, F.; Buruiana, A.-T.; Galca, A.-C.; Becherescu, N.; Burducea, I.; Mihai, C.; Velea, A. Influence of Deposition Method on the Structural and Optical Properties of Ge2Sb2Te5. *Materials* **2021**, *14*, 3663. https://doi.org/10.3390/ma14133663

Academic Editor: Rainer Hippler

Received: 11 June 2021
Accepted: 27 June 2021
Published: 30 June 2021

Publisher's Note: MDPI stays neutral with regard to jurisdictional claims in published maps and institutional affiliations.

Copyright: © 2021 by the authors. Licensee MDPI, Basel, Switzerland. This article is an open access article distributed under the terms and conditions of the Creative Commons Attribution (CC BY) license (http://creativecommons.org/licenses/by/4.0/).

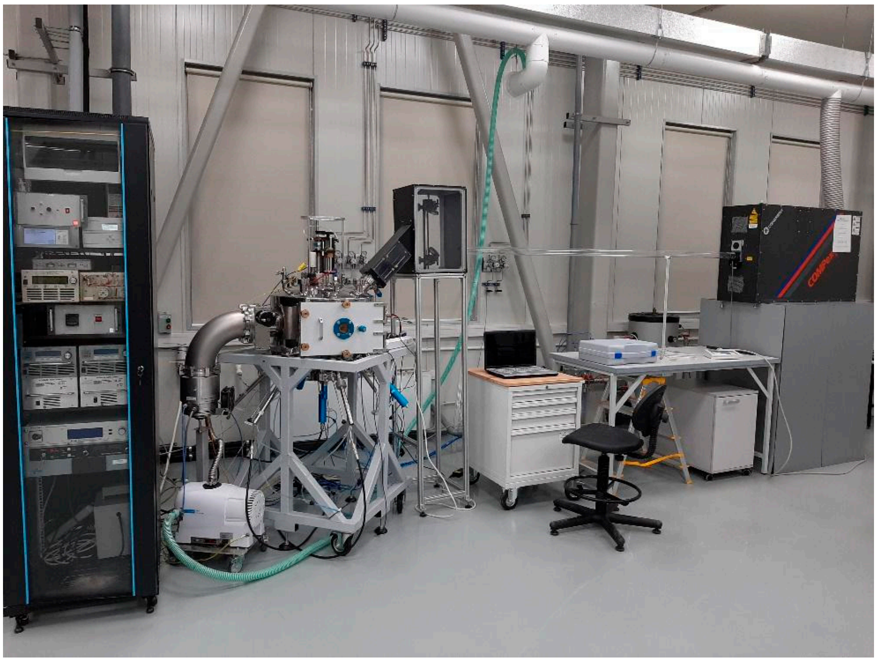

Figure S1. The custom built MSPLD deposition system.
